# Pediatric Endoscopy During COVID-19 Times

**DOI:** 10.3389/fped.2021.750717

**Published:** 2021-12-16

**Authors:** Ron Shaoul, Andrew S. Day

**Affiliations:** ^1^Rambam Medical Center, Faculty of Medicine, Pediatric Gastroenterology and Nutrition Institute, Ruth Rappaport Children's Hospital of Haifa, Haifa, Israel; ^2^Department of Paediatrics, University of Otago Christchurch, Christchurch, New Zealand

**Keywords:** endoscopy, children, pediatrics, COVID-19, healthcare

## Abstract

The global COVID-19 pandemic has led to healthcare resources being diverted or stretched, especially during periods of lock-down in affected countries. Disruptions to normal services have resulted in reduced or delayed provision of endoscopy in many countries, with consequent impacts on diagnosis or management of digestive diseases and upon endoscopy training. This review article aims to highlight key aspects of the impact of the pandemic upon endoscopy services, with a focus upon endoscopy in children.

## Introduction

Severe acute respiratory coronavirus (SARS-CoV)-2 is a member of the *Coronaviridae* family: this family of viruses includes a number of virulent strains known to infect animals and humans (1). The disease caused by SARS-CoV-2, known as coronavirus disease (COVID)-19, was first noted in 2019. Subsequently, the World Health Organization (WHO) declared COVID-19 to be a pandemic on March 11, 2020 (2). Since then, the COVID-19 pandemic has spread worldwide with more than 250 million laboratory-confirmed cases and more than 5 million deaths as of November 20th, 2021, (https://www.worldometers.info/coronavirus/) leading to widespread social and economic disruption.

Soon after the WHO declaration in March 2020, numerous health authorities across different countries (for e.g., the US Surgeon General, the American College of Surgeons, the Centers for Disease Control and Prevention, and Chief Medical Officers across Canada) advised medical practitioners to suspend all elective medical procedures (3). This decision affected surgeons, gastroenterologists and other disciplines (4). Consequently, endoscopy centers have undergone significant changes based on international and local guidelines and have taken significant and unprecedented steps to avoid transmission of the virus (4).

While some early reports suggested that there was a low risk of COVID-19 transmission consequent to GI endoscopy (5), other reports indicated a high prevalence of SARS-CoV-2 infection in patients scheduled for digestive endoscopy (6). These contradictory reports resulted in confusion and uncertainty in decision making.

Although the initial force of the pandemic has passed in most countries, there remain ongoing waves of infection with particular impact in some parts of the world. Further, this impact will have ongoing repercussions for some time. This review focuses on the impact of the COVID-19 pandemic upon endoscopy services, focusing on pediatric services where available, highlighting the immediate effects in 2020 and some of the ongoing consequences thereafter.

## Methods

A literature search was performed using MEDLINE, Pubmed, and the Cochrane Library with the last search date of November 1st 2021. Search terms included COVID-19, endoscopy, children, pediatric, guidelines “SARS-CoV-2,” “gastrointestinal endoscopy,” and “digestive system endoscopy.”

### Severe Acute Respiratory Coronavirus (SARS-CoV)-2 and COVID-19

SARS-CoV-2 can spread from person to person by contact, airborne and droplet routes (7). It behaves as an opportunistic airborne pathogen following a cough or during procedures that generate aerosols (3). van Doremalen et al. (8) showed ongoing detection of the virus in aerosols for up to 3 h. Aerosols can also be transmitted for several meters (9). Furthermore, the virus can persist on surfaces, such as on items in the rooms of patients with active COVID-19 (9).

Children of any age can be infected by SARS-CoV-2, but is more commonly recognized in older children and adolescents. Overall, pediatric cases of COVID-19 are thought to represent <5% of total cases (10). However, the true rate of infection in children is likely much higher than this. Testing in many studies was undertaken only in symptomatic individuals or those who were hospitalized (11). This is relevant given that many children infected with COVID-19 are asymptomatic or have mild symptoms, such as fever, cough, gastrointestinal symptoms, pharyngitis, or changes in sense of smell or taste (11). In addition, recent data suggests that children are less likely to become infected after contact with someone who is infected with SARS-CoV-2 (11).

Recent reports demonstrate that children and adolescents infected with the virus have similar viral loads to adults (12, 13): consequently, they are just as likely to transmit the virus to others (14). In addition, the viral load may be unrelated to the presence or absence of symptoms in that individual (15, 16). For example, Sola et al. (17) studied the pattern of positive SARS-CoV-2 test results in 33,041 asymptomatic children presenting for surgical or medical care at 28 children's hospitals across the United States of America (USA). Two hundred and fifty of these children (ranging in age between 0 and 18 years) had positive SARS-CoV-2 tests.

The overall pooled prevalence was 0.65% (95% CI, 0.47–0.83%), with rates varying from 0 to 2.2% between centers.

### Transmission of SARS-CoV-2 During GI Endoscopy

The characteristics of the SARS-CoV-2 virus mean that an endoscopic procedure is a potential mechanism for spreading the infection. Person-to-person spread, respiratory droplets, generation of aerosols, and direct contact with contaminated surroundings or body fluids are all relevant to endoscopy (18).

Keil et al. (19) studied spread of the virus using an experimental model of endoscopic procedures in a specialized laboratory. This model evaluated the formation and movement of potentially infectious fluid particles from the patient's body to the environment via the endoscope. They found liquid coming through the working channel of the endoscope with biopsy forceps or other instruments generates droplets with a diameter in the range of 0.1–1.1 mm and an initial velocity of up to 0.9 m/s. They developed a protective cover that completely eliminated droplet spread (19).

In addition to the concern about spread by respiratory secretions, there is also an increasing concern about the potential for fecal-oral spread. The high frequency of gastrointestinal symptoms in individuals with COVID-19, the localization of angiotensin converting enzyme 2 in the intestinal mucosa and the identification of active viral particles in stool for prolonged periods after infection highlight these concerns (20, 21).

Although there are significant concerns about the risk of spread of the virus from an infected patient to endoscopy staff, the overall risk if appropriate precautions are followed appear to be low (22). In addition to the patient-related processes mentioned earlier, other suggested steps within the endoscopy unit include the use of personal protective equipment, limitations on the number of staff in the endoscopy suite and procedural modifications.

Papanikolaou et al. (23) conducted a multicenter study to assess the risk of infection with an endoscopic procedure. In the setting of low risk or negative COVID-19 testing in 1,135 individuals, 254 were tested after their procedure and eight were shown to be positive. Amongst 163 endoscopic personnel assessed in this report, five tested positive during the study period.

### Guidelines for the Undertaking of Endoscopy in the Setting of COVID-19

Guidelines have been issued by several adult societies (24–26) and one pediatric society (3). These guidelines concentrate on four main themes: (a) How to perform endoscopic procedures during the COVID-19 pandemic, (b) Which endoscopic procedures should always be done and which should be deferred? (Tables 1, 2), (c) How to protect endoscopy unit staff during the pandemic? and (d) What are the gaps in current knowledge and what is still required in this rapidly evolving field?

**Table 1:** Patients considered high risk for endoscopic procedures [adapted from Sinonquel et al. (27)].

1. Patients with symptoms suggestive of an upper respiratory infection or with an elevated temperature of ≥37.5°C.
2. Patients with a history of close contact with more or more individuals with established or suspected COVID-19 within the last fortnight.
3. Patients with a personal history of travel to area(s) with pandemic COVID-19 within the last fortnight. Including any new or emerging “hot spots.”
4. Patients reporting significant general fatigue and/or shortness of breath.
5. Patients complaining of dysosmia and/or dysgeusia (loss of smell or taste) without any clear underlying cause.
6. Patients complaining of GI symptoms (including diarrhea) lasting for at least 4 days without a clear identifiable cause.

**Table 2:** Conditions that may need urgent endoscopy regardless of COVID status.

•Bleeding (or suspected bleeding) from the upper/lower gastrointestinal tract
•Cholangitis requiring endoscopic retrograde cholangiopancreatography
•Symptomatic bile duct/pancreatic disease
•Endoscopic release of a gastrointestinal stricture
•GI cancer requiring early treatment
•Other conditions requiring urgent treatment based on the decision by the facility

Initially, the AGA recommended that endoscopy units may recommence doing elective endoscopic procedures when the number of new cases of COVID-19 in the local area has reduced consistently for at least a fortnight (American Gastroenterological Association, Digestive Health Physicians Association. AGA/DHPA Joint Guidance for Resumption of Elective Endoscopy. Available at: https://gastro.org/news/aga-dhpa-release-guidance-forresuming-elective-endoscopy/). The time of resumption of endoscopy services must also consider other local or national advice, local resources and the ability to provide a safe environment for patients and staff.

The AGA guide specified that scheduled endoscopies should still be prioritized by level of urgency, depending upon individual patient considerations and physician professional judgement. They also stated that, wherever possible, all patients should be tested for active COVID-19 infection with PCR-based testing within 48 h prior to their endoscopy. Subsequently, the AGA Institute recommended not undertaking pretesting in “high” prevalence or “low” prevalence (< 0.5%) areas, due to the possibility of false negative results in the former and false-positive results in the latter areas (28).

The position paper presented by the North American Society for Pediatric Gastroenterology, Hepatology, and Nutrition (NASPGHAN) recommended that all endoscopic procedures in children should be performed in a negative pressure room with all personnel following strict airborne, contact and droplet precautions regardless of the individual risk category of the patient (3). These precautions (i.e., enhanced PPE) include using a standard filtering facepiece respirator (such as N95, N99, FFP2/3, or PAPR), two pairs of gloves, full facial protection (either a visor or a face shield), a hairnet, full body water-resistant disposable gown or coveralls and appropriate shoe covers.

### Practical Considerations Prior to Undertaking an Endoscopic Procedure

The exact processes to be considered prior to endoscopy may vary according to current local requirements or restrictions. Factors such as the current number of community cases and the vaccination status of the individual are relevant. In addition, the clinical indication for the endoscopic procedure needs to be assessed.

In general terms, however, pre-admission screening conducted by telephone or video consultation is highly recommended (27). Before arrival, patients should be asked the following questions: (1) recent or current fever, (2) recent travel history (especially travel to any country or region with a high incidence of COVID-19 transmission within the last 2 weeks), (3) history of contact with anyone who has respiratory or general symptoms (in the last 14 days) and (4) any clustering. If one or more of these risk factors is present, the patient should be considered to be a suspected case and COVID-19 RT-PCR testing (e.g., nasopharyngeal swab) should be conducted prior to undertaking the endoscopic procedure.

Before entering the waiting room, patients should have measurement of their temperature and the above questions should be asked again. Local social distancing rules should be strictly applied in the waiting area: this may include the use of face masks and appropriate spacing of chairs (27). Children should be accompanied by a maximum of one parent.

In addition, before any clinical examination the attending physician should again ask the patient about any systemic or respiratory symptoms that could be suggestive of active COVID-19 (27). If a patient has a positive or inconclusive RT-PCR test, the procedure should be considered high risk and should only be conducted if the clinical situation demands (27). Furthermore, if a patient has current symptoms or is otherwise at high risk for current infection (Table 1), but has such an urgent indication for endoscopy that there is inadequate time to have a nasopharyngeal swab, then their procedure should also be considered high risk (6).

Any endoluminal procedures (such as diagnostic or therapeutic endoscopy or even placement of feeding devices) should be considered potentially high-risk. Before commencing any procedure involving the GI tract it is important to fully assess the necessity and urgency of the procedure. Assessment of the presence of alarm symptoms such as involuntary weight loss, dysphagia, bleeding or obstructive jaundice is highly important (Table 2) (24, 27).

### Implications of Pre-procedural COVID-19 Polymerase Chain Reaction Testing on Routine Endoscopic Practice

Forde et al. (29) performed 396 PCR tests in patients prior to endoscopy in April and May 2020 in one North American centre. Only one of these patients had a positive PCR result, providing a positive test rate of 0.25% (95% confidence interval, 0.01–1.4%). None of the patients who denied any suggestive symptoms on initial screening and had a negative PCR test failed their immediate pre-procedure questionnaire or body temperature check on the day of procedure. Furthermore, none of the endoscopy staff involved in the management of these patients were diagnosed with COVID-19 or developed symptoms suggestive of infection. The authors concluded that screening questionnaires are an effective tool for the identification of patients at greater risk of infection who should have deferral of their procedure.

Bowyer et al. (30) retrospectively reviewed pre-procedural screening evaluations in a group of 1,000 patients who were scheduled to have an elective outpatient endoscopic procedure between May 22 and June 28, 2020. Key data included demographics, symptoms as reported in the American Society for Gastrointestinal Endoscopy (ASGE) COVID-19 risk preprocedural screening questionnaire, and the results of PCR testing. Eight of the 1,000 patients had positive COVID-19 tests: three of them had reported at least one symptom on the risk screening questionnaire. In addition, 119 patients reported symptoms on the risk screening questionnaire but had negative COVID-19 tests. This assessment of the ASGE COVID-19 risk screening questionnaire provided positive and negative predictive values of 2.46 and 99.43%, respectively. The authors concluded that symptom-based screening alone was not sufficient as a primary preprocedural assessment tool during the COVID-19 pandemic.

Khorrami Minaei et al. (31) performed a retrospective review to assess the usefulness of clinical screening and pre-procedure PCR testing for the identification of high risk patients for SARS-CoV-2 infection. They included a consecutive cohort of 361 patients undergoing endoscopy at a tertiary teaching hospital between 22nd April and 22nd June 2020. Clinical screening, following a defined protocol, detected 13 patients with a high risk of infection (3.6%, 95% CI 2.62–4.58) while the pre-procedure PCR test was positive in five patients (1.40% 95% CI 0.20–2.60). Three patients developed COVID-19 and one died from the disease. Agreement between both strategies was poor, with a Kappa value of 0.093 (95% CI 0.001–0.185). Clinical screening only identified one of the five patients with a positive PCR test. The authors concluded that clinical screening prior to endoscopy has poor agreement with pre-procedure PCR testing.

Say et al. (32) suggested that patient risk stratification before endoscopy should be performed based on symptoms and sick contacts if PCR testing for infection was not available. The authors recommended that urgent procedures should be prioritized. Examples included the removal of foreign bodies, evaluation of gastrointestinal bleeding, and procedures in hospitalized patients. The authors further recommended only performing “essential” procedures in outpatients: these were defined as procedures that would lead to significant patient harm if they were delayed for >2–3 months. Overall, the process to finalize the scheduling and timing of an endoscopic should include discussions and shared decision-making involving the endoscopist, the patient, and the patient's family.

### Endoscopic Procedures Involving Patients With Inflammatory Bowel Disease During the COVID-19 Pandemic

Endoscopy is mandatory for patients with a high suspicion of IBD. In patients with a clinical suspicion of Crohn's disease who have a normal endoscopy, visualization of the small intestine is also required. Iacucci et al. (33) addressed the issue of endoscopy in patients with IBD. The authors suggested that in addition to excluding SARS-CoV-2, exclusion of other gastrointestinal infections is also important. They suggested using detailed history and the results of biomarkers to differentiate between IBD and IBS whenever possible.

Iacucci et al. (33) suggested postponing endoscopic assessments in patients who have only mild abnormalities on blood tests and fecal calprotectin (33). In the setting of acute severe exacerbation of known ulcerative colitis they suggested that flexible sigmoidoscopy (at least) should be undertaken if the patient's last colonoscopy was more than 3 months earlier. This assessment would be considered essential to confirm the clinical findings, to define the current extent and severity of disease and to exclude concurrent GI infections (such as cytomegalovirus).

In a similar fashion, Turner et al. (34) also recommended non-urgent endoscopy in children with IBD should be postponed during the pandemic.

### Worldwide Impact of COVID-19 Upon Adult and Pediatric Endoscopic Practice

Several authors have assessed the impact of the pandemic upon endoscopy practice. Ruan et al. (35) performed an international survey of pediatric endoscopists across 27 countries. Most of the 145 respondents reported that elective procedures were postponed with a reduction in activity to <10% of usual activity. Although almost all the units were undertaking emergency or urgent endoscopy procedures, only half of the units employed specific guidelines to determine urgency. Furthermore, the units of the respondents differed widely in the use of screening or testing guidelines.

These findings were similar to those reported in adult centers. Forbes et al. (36) conducted a web-based survey of the impact upon endoscopic activity in North American centers in March and April 2020. Two-thirds of the 73 respondents reported a 90% reduction in endoscopy volume. These findings were extended internationally in a web-based survey of endoscopy activity across 252 adult gastroenterology units in 55 countries (37). This study, conducted in April and May 2020, showed a mean reduction of 83% in endoscopy volume across all areas of the world except Australasia, where activity was maintained at ~40% of normal activity.

Recent studies have demonstrated this impact in specific countries. Issaka et al. (38) retrospectively examined delays in endoscopic procedures in one centre in USA. In the cohort of 480 patients, colonoscopy was most frequently delayed (49%), especially in the setting of colorectal cancer screening. At the time the report was written, less than half of the delayed procedures had been completed, with the diagnosis of 12 cancers eventually made. The median time of delay in those who had undergone their planned procedure was 88 days.

A study conducted in one geographical area of Korea illustrated further impact on endoscopic procedures (39). This retrospective study was conducted in Daegu, a region of Korea with a high case burden at that time. Three hundred and thirty-six emergency procedures conducted over a five and half week period in February and March 2020 were evaluated. The number of cases was less than half of the procedures conducted in the same unit during the same periods in both 2018 and 2019. The patients who were endoscoped in 2020 appeared to be sicker (with a lower hemoglobin), were twice as likely to have endoscopic abnormalities seen and almost five times as likely to need an endoscopic intervention.

These findings appeared to indicate that the triage process at this location enabled the identification of more severe patients and that less severe patients were deferred. The authors did not present any data on adverse outcomes on patients were not triaged to undergo a procedure. They did, however, report that there were no cases of transmission from patients to hospital or endoscopy staff.

### Worldwide Impact of COVID-19 Upon Adult and Pediatric Endoscopic Practice

Consequent to the dramatic reduction in endoscopy volume and alterations in patient selection, significant impact upon endoscopy training has also been noted (40, 41). Recently, Shin et al. (42) evaluated the impact upon training for GI fellows in Korea, using a web-based questionnaire. More than half of the 94 respondents noted a reduction in endoscopy sessions and volume, with 78.9% reporting concern about their education and training.

The impact on endoscopy for pediatric gastroenterology fellow training in North America was assessed in a short survey in April 2020 (43). Fellows ceased involvement in endoscopy procedures in 26 of 51 programs that gave replies to the survey. The survey did not assess the impact upon endoscopy procedures over a longer time period.

Nita et al. (44) surveyed 144 young members of ESPGHAN in mid-2020. The COVID-19 pandemic almost universally resulted in adverse impacts upon the endoscopy practice of the respondents. In particular, 82 of the respondents mentioned restrictions to semi-urgent or emergency endoscopy procedures.

One consequence of the pandemic is a move from face-to-face to virtual interactions when feasible. This has also occurred in the setting of trainee interviews (45). An assessment of satisfaction with this mode of interviewing showed generally high acceptance and satisfaction.

The changes in endoscopy practice have also resulted in the development of innovative technology. These include new shielding devices (46) and even the use of robotic endoscopic processes (47). More recently, Furukawa et al. (48) described their development of a surgical mask designed to prevent spread of droplets from a patient undergoing an upper endoscopic procedure. The authors demonstrated a marked reduction in droplets using specialized imaging technique. Other novel devices include the Endoprotector (49), the C-Cube (50), the ORIGAMI face shield (51) and an endoscopic shield (52). These innovations have not been evaluated specifically in children.

### Ongoing Repercussions of COVID-19-Related Changes in Endoscopic Activity

Even though the first waves of the pandemic have passed, the repercussions persist. Ho et al. (53) attempted to predict the recovery of disrupted endoscopy activity in England using national data from prior to the pandemic to the end of October 2020. The analysis indicated that there would be a backlog of almost 500,000 endoscopic procedures by January 2021. The authors further predicted that elimination of this backlog would take until mid-2022 even with an increase in capacity to 130% of normal. Further and ongoing interruptions would only exacerbate this impact and delay catch-up substantially.

## Conclusions

The COVID-19 pandemic has had a global adverse effect on humanity, with significant morbidity and mortality. While healthcare services have been greatly impacted overall, due to diversion of resources to care for those diagnosed with COVID-19, there have also been great impacts on endoscopy procedures. The predicted flow-on effects of a reduction in endoscopic activity have included consequences such as longer diagnostic delay and reduced training opportunities.

Much of the data available reflects regional and time-related differences in infection rates and consequent variations in practice. As the effects of the waves of infection wax and wane, so to will the direct impacts. These features of the pandemic do impact on the way that endoscopy services are conducted in various locations and countries. None-the-less, depending on the course of the COVID-19 pandemic over the coming months, there will likely be further impacts on endoscopy services.

## Author Contributions

RS and AD contributed to all aspects of the preparation of this manuscript and have reviewed and approved the final version of the manuscript. Both authors contributed to the article and approved the submitted version.

## Conflict of Interest

The authors declare that the research was conducted in the absence of any commercial or financial relationships that could be construed as a potential conflict of interest.

## Publisher's Note

All claims expressed in this article are solely those of the authors and do not necessarily represent those of their affiliated organizations, or those of the publisher, the editors and the reviewers. Any product that may be evaluated in this article, or claim that may be made by its manufacturer, is not guaranteed or endorsed by the publisher.

## References

[1] Dai L Gao GF. Viral targets for vaccines against COVID-19. Nat Rev Immunol. (2021) 21:73–82. 10.1038/s41577-020-00480-0 33340022 PMC7747004

[2] Bedford J Enria D Giesecke J Heymann DL Ihekweazu C Kobinger G. COVID-19: towards controlling of a pandemic. Lancet. (2020) 395:1015–18. 10.1016/S0140-6736(20)30673-5 32197103 PMC7270596

[3] Walsh CM Fishman DS Lerner DG. Pediatric endoscopy in the era of coronavirus disease 2019: A North American society for pediatric gastroenterology, hepatology, and nutrition position paper. J Pediatr Gastroenterol Nutr. (2020) 70:741–50. 10.1097/MPG.0000000000002750 32443022 PMC7273958

[4] Perisetti A Goyal H Sharma N. Gastrointestinal endoscopy in the Era of COVID-19. Front Med. (2020) 7:587602. 10.3389/fmed.2020.587602 33330546 PMC7732601

[5] Repici A Aragona G Cengia G Cantù P Spadaccini M Maselli R. Low risk of COVID-19 transmission in GI endoscopy. Gut. (2020) 69:1925–27. 10.1136/gutjnl-2020-321341 32321857

[6] Pamplona J Solano R Ramírez M Durandez R Mohamed F Pardo L. High prevalence of SARS-CoV-2 infection in patients scheduled for digestive endoscopy after the peak of the first wave of the pandemic. Gastroenterol Hepatol. (2021) 44:614–9. 10.1016/j.gastre.2021.03.002 33862154 PMC8056966

[7] Li Q Guan X Wu P Wang X Zhou L Tong Y. Early transmission dynamics in Wuhan, China, of novel coronavirus-infected pneumonia. N Engl J Med. (2020) 382:1199–207. 10.1056/NEJMoa2001316 31995857 PMC7121484

[8] van Doremalen N Bushmaker T Morris DH Holbrook MG Gamble A Williamson BN. Aerosol and surface stability of SARS-CoV-2 as compared with SARS-CoV-1. N Engl J Med. (2020) 382:1564–67. 10.1056/NEJMc2004973 32182409 PMC7121658

[9] Santarpia JL Rivera DN Herrera VL Morwitzer MJ Creager HM Santarpia GW. Aerosol and surface contamination of SARS-CoV-2 observed in quarantine and isolation care. Sci Rep. (2020) 10:12732. 10.1038/s41598-020-69286-3 32728118 PMC7391640

[10] Jurado Hernández JL Álvarez Orozco IF. COVID-19 in children: respiratory involvement and some differences with the adults. Front Pediatr. (2021) 9:622240. 10.3389/fped.2021.622240 33855003 PMC8039144

[11] Zimmermann P Curtis N. Why is COVID-19 less severe in children? A review of the proposed mechanisms underlying the age-related difference in severity of SARS-CoV-2 infections. Arch Dis Child. (2021) 106:429–39. 10.1136/archdischild-2020-320338 33262177

[12] Jones TC Mühlemann B Veith T Biele G Zuchowski M Hofmann J. An analysis of SARS-CoV-2 viral load by patient age. medRxiv. (2020) 2020:2020.06.08.20125484. 10.1101/2020.06.08.20125484

[13] Baggio S L'Huillier AG Yerly S Bellon M Wagner N Rohr M. SARS-CoV-2 viral load in the upper respiratory tract of children and adults with early acute COVID-19. Clin Infect Dis. (2020) 73:148–50. 10.1101/2020.07.17.20155333 32761228 PMC7454380

[14] Park YJ Choe YJ Park O Park SY Kim YM Kim J. Contact tracing during coronavirus disease outbreak, South Korea, (2020). Emerg Infect Dis. (2020) 26:2465–68. 10.3201/eid2610.201315 32673193 PMC7510731

[15] Lee S Kim T Lee E Lee C Kim H Rhee H. Clinical course and molecular viral shedding among asymptomatic and symptomatic patients with SARS-CoV-2 infection in a community treatment center in the Republic of Korea. JAMA Intern Med. (2020) 180:1–6. 10.1001/jamainternmed.2020.3862 32780793 PMC7411944

[16] Hurst JH Heston SM Chambers HN Cunningham HM Price MJ Suarez L. SARS-CoV-2 infections among children in the biospecimens from respiratory virus-exposed kids (BRAVE Kids) study. Clin Infect Dis. (2021) 73:2875–82. 10.1101/2020.08.18.20166835 33141180 PMC7665428

[17] Sola AM David AP Rosbe KW Baba A Ramirez-Avila L Chan DK. Prevalence of SARS-CoV-2 infection in children without symptoms of coronavirus disease 2019. JAMA Pediatr. (2021) 175:198–201. 10.1001/jamapediatrics.2020.4095 32840605 PMC7851725

[18] Soetikno R Teoh AYB Kaltenbach T Lau JYW Asokkumar R Cabral-Prodigalidad P. Considerations in performing endoscopy during the COVID-19 pandemic. Gastrointest Endosc. (2020) 92:176–83. 10.1016/j.gie.2020.03.3758 32229131 PMC7195289

[19] Keil R Hlava Š Stanovský P Zdimal V Sovicek J Trojanek M. Commonly available but highly effective protection against SARS-CoV-2 during gastrointestinal endoscopies. PLoS ONE. (2021) 16:e0254979. 10.1371/journal.pone.0254979 34297736 PMC8301622

[20] Scaldaferri F Ianiro G Privitera G Lopetuso LR Vetrone LM Petito V. The thrilling journey of SARS-CoV-2 into the intestine: from pathogenesis to future clinical implications. Inflamm Bowel Dis. (2020) 26:1306–14. 10.1093/ibd/izaa181 32720978 PMC7454647

[21] Neurath MF Überla K Ng SC. Gut as viral reservoir: lessons from gut viromes, HIV and COVID-19. Gut. (2021) 70:1605–8. 10.1136/gutjnl-2021-324622 33903146

[22] Lui RN. Safety in endoscopy for patients and healthcare workers during the COVID-19 pandemic. Tech Innov Gastrointest Endosc. (2021) 23:170–78. 10.1016/j.tige.2020.10.004 33103130 PMC7568769

[23] Papanikolaou IS Tziatzios G Chatzidakis A Facciorusso A Crino SF Gkolfakis P. COVID-19 in the endoscopy unit: How likely is transmission of infection? Results from an international, multicenter study. World J Gastrointest Endosc. (2021) 13:416–25. 10.4253/wjge.v13.i9.416 34630891 PMC8474700

[24] Irisawa A Furuta T Matsumoto T Kawai T Inaba T Kanno A. Gastrointestinal endoscopy in the era of the acute pandemic of coronavirus disease 2019: Recommendations by Japan Gastroenterological Endoscopy Society (Issued on April 9th, 2020). Dig Endosc. (2020) 32:648–50. 10.1111/den.13703 32335946 PMC7267159

[25] Chai N Mei Z Zhang W Du C Wang X Li L. Endoscopy works during the pandemic of coronavirus COVID-19: recommendations by the Chinese Society of Digestive Endoscopy. United Eur Gastroenterol J. (2020) 8:798–803. 10.1177/2050640620930632 32615871 PMC7335943

[26] Gralnek IM Hassan C Beilenhoff U Antonelli G Ebigbo A Pellisè M. ESGE and ESGENA position statement on gastrointestinal endoscopy and the COVID-19 pandemic. Endoscopy. (2020) 52:483–90. 10.1055/a-1155-6229 32303090 PMC7295280

[27] Sinonquel P Roelandt P Demedts I Van Gerven L Vandenbriele C Wilmer A. COVID-19 and gastrointestinal endoscopy: What should be taken into account? Dig Endosc. (2020) 32:723–31. 10.1111/den.13706 32335962 PMC7267439

[28] Sultan S Siddique SM Altayar O Caliendo AM Davitkov P Feuerstein JD. AGA Institute rapid review and recommendations on the role of pre-procedure SARS-CoV-2 testing and endoscopy. Gastroenterology. (2020) 159:1935–48.e5. 10.1053/j.gastro.2020.07.043 32735862 PMC7386603

[29] Forde JJ Goldberg D Sussman D Soriano F Barkin JA Amin S. Yield and implications of pre-procedural COVID-19 polymerase chain reaction testing on routine endoscopic practice. Gastroenterology. (2020) 159:1538–40. 10.1053/j.gastro.2020.05.062 32464146 PMC7255132

[30] Bowyer B Thukral C Patel S Dovalovsky K Bowyer SG Ford J. Outcomes of symptom screening and universal COVID-19 reverse transcriptase polymerase chain reaction testing before endoscopy in a community-based ambulatory surgery center. Gastrointest Endosc. (2021) 93:1060–64.e1. 10.1016/j.gie.2020.10.001 33039400 PMC7543774

[31] Khorrami Minaei S Garrido Durán C García Hernández M García Amengual I Mena Ribas A. Poor agreement between clinical screening and universal pre-procedure SARS-CoV-2 PCR testing prior to endoscopy. Rev Esp Enferm Dig. (2021) 113:649–55. 10.17235/reed.2021.7612/2020 33588573

[32] Say DS de Lorimier A Lammers CR Natale J Lakshminrusimha S Wiedeman J. Risk stratification and personal protective equipment use in pediatric endoscopy during the coronavirus disease 2019. Outbreak: a single-center protocol. J Pediatr Gastroenterol Nutr. (2020) 70:751–54. 10.1097/MPG.0000000000002731 32443023 PMC7273944

[33] Iacucci M Cannatelli R Labarile N Mao R Panaccione R Danese S. Endoscopy in inflammatory bowel diseases during the COVID-19 pandemic and post-pandemic period. Lancet Gastroenterol Hepatol. (2020) 5:598–606. 10.1016/S2468-1253(20)30119-9 32305075 PMC7162648

[34] Turner D Huang Y Martín-de-Carpi J Aloi M Focht G Kang B. Corona virus disease 2019 and paediatric inflammatory bowel diseases: global experience and provisional guidance (March 2020) from the paediatric IBD porto group of European society of paediatric gastroenterology, hepatology, and nutrition. J Pediatr Gastroenterol Nutr. (2020) 70:727–33. 10.1097/MPG.0000000000002729 32443020 PMC7273950

[35] Ruan W Fishman DS Lerner DG Engevik MA Elmunzer BJ Walsh CM. Changes in pediatric endoscopic practice during the coronavirus disease 2019 pandemic: results from an international survey. Gastroenterology. (2020) 159:1547–50. 10.1053/j.gastro.2020.05.068 32485178 PMC7260478

[36] Forbes N Smith ZL Spitzer RL Keswani RN Wani SB Elmunzer BJ. Changes in gastroenterology and endoscopy practices in response to the coronavirus disease 2019 pandemic: results from a North American survey. Gastroenterology. (2020) 159:772–74.e13. 10.1053/j.gastro.2020.04.071 32376410 PMC7196540

[37] Parasa S Reddy N Faigel DO Repici A Emura F Sharma P. Global impact of the COVID-19 pandemic on endoscopy: an international survey of 252 centers From 55 countries. Gastroenterology. (2020) 159:1579–81.e5. 10.1053/j.gastro.2020.06.009 32534934 PMC7289081

[38] Issaka RB Feld LD Kao J Hegarty E Snailer B Kalra G. Real-world data on the impact of COVID-19 on endoscopic procedural delays. Clin Transl Gastroenterol. (2021) 12:e00365. 10.14309/ctg.0000000000000365 34060496 PMC8162484

[39] Kim KH Kim SB Kim TN. Changes in endoscopic patterns before and during COVID-19 outbreak: Experience at a single tertiary center in Korean. World J Clin Cases. (2021) 9:3576–85. 10.12998/wjcc.v9.i15.3576 34046457 PMC8130062

[40] Pawlak KM Kral J Khan R Amin S Bilal M Lui RN. Impact of COVID-19 on endoscopy trainees: an international survey. Gastrointest Endosc. (2020) 92:925–35. 10.1016/j.gie.2020.06.010 32535193 PMC7287420

[41] Marasco G Nardone OM Maida M Boskoski I Pastorello L Scaldaferri F. Impact of COVID-19 outbreak on clinical practice and training of young gastroenterologists: A European survey. Dig Liver Dis. (2020) 52:1396–402. 10.1016/j.dld.2020.05.023 32507619 PMC7245276

[42] Shin HP Cha JM Kim BK Myung D-S Moon S-H Song MJ. [Impact of COVID-19 on gastroenterology fellowship training]. Korean J Gastroenterol. (2021) 77:205–13. 10.4166/kjg.2021.049 34035196 PMC12286485

[43] Mallon D Pohl JF Phatak UP Fernandes M Rosen JM Lusman SS. Impact of COVID-19 on pediatric gastroenterology fellow training in North America. J Pediatr Gastroenterol Nutr. (2020) 71:6–11. 10.1097/MPG.0000000000002768 32369320 PMC7273936

[44] Nita AF Tsita D Grima AM Cameron F Rock NM Tapsas D. Understanding and responding to the impact of COVID-19 on paediatric gastroenterology training & practice of young ESPGHAN members. J Pediatr Gastroenterol Nutr. (2021) 73:592–8. 10.1097/MPG.0000000000003239 34269327 PMC8527914

[45] Kamboj AK Raffals LE Martin JA Chandrasekhara V. Virtual interviews during the COVID-19 pandemic: a survey of advanced endoscopy fellowship applicants and programs. Tech Innov Gastrointest Endosc. (2021) 23:159–68. 10.1016/j.tige.2021.02.001 34697608 PMC8528488

[46] Kikuchi D Suzuki Y Hoteya S. Shielding method for the endoscopic procedures during the COVID-19 pandemic. Dig Endosc. (2020) 32:e160–61. 10.1111/den.13821 33020948 PMC7675636

[47] Onaizah O Koszowska Z Winters C Subramanian V Jayne D Arezzo A. Guidelines for robotic flexible endoscopy at the time of COVID-19. Front Robot AI. (2021) 8:612852. 10.3389/frobt.2021.612852 33718439 PMC7947201

[48] Furukawa K Sato K Okachi S Kawashima H Fujishiro M. Surgical mask designed for endoscopic procedures to prevent the diffusion of droplets. Acta Gastroenterol Belg. (2021) 84:517–19. 10.51821/84.3.024 34599580

[49] Campos S Carreira C Marques PP Vieira A. Endoprotector: Protective box for safe endoscopy use during COVID-19 outbreak. Endosc Int Open. (2020) 8:E817–21. 10.1055/a-1180-8527 32509960 PMC7266658

[50] Traina M Amata M Granata A Ligresti D Gaetano B. The C-Cube: an endoscopic solution in the time of COVID-19. Endoscopy. (2020) 52:E351–52. 10.1055/a-1190-3462 32559785 PMC7516370

[51] Onoyama T Fujii M Isomoto H. Useful face-protective shield “ORIGAMI” for gastrointestinal endoscopy during the COVID-19 pandemic. Dig Endosc. (2020) 32:998. 10.1111/den.13780 32578910 PMC7362137

[52] Sagami R Nishikiori H Sato T Murakami K. Endoscopic shield: barrier enclosure during the endoscopy to prevent aerosol droplets during the COVID-19 pandemic. VideoGIE. (2020) 5:445–48. 10.1016/j.vgie.2020.05.002 32395674 PMC7211574

[53] Ho KMA Banerjee A Lawler M Rutter MD Lovat LB. Predicting endoscopic activity recovery in England after COVID-19: a national analysis. Lancet Gastroenterol Hepatol. (2021) 6:381–90. 10.1016/S2468-1253(21)00058-3 33713606 PMC7946568

